# Chlorpyrifos induces spermatogenic dysfunction via ferroptosis in Sertoli cells

**DOI:** 10.1016/j.gendis.2025.101601

**Published:** 2025-03-14

**Authors:** Yan Fu, Xu Huang, Siyuan Wang, Qitong Guo, Yuhao Wu, Xiangqin Zheng, Junke Wang, Shengde Wu, Lianju Shen, Guanghui Wei

**Affiliations:** a Department of Urology, Children's Hospital of Chongqing Medical University, Chongqing 400014, China; b Pediatric Research Institute, Chongqing Key Laboratory of Structural Birth Defect and Reconstruction, Ministry of Education Key Laboratory of Child Development and Disorders, National Clinical Research Center for Child Health and Disorders, Children's Hospital of Chongqing Medical University, Chongqing 400014, China; c Department of Cardiothoracic Surgery, Children's Hospital of Chongqing Medical University, Chongqing 400014, China; d Department of Urology, The Second Affiliated Hospital of Chongqing Medical University, Chongqing 400010, China

**Keywords:** Blood-testis barrier, Clockophagy, CPF, Ferroptosis, Sertoli cells

## Abstract

Chlorpyrifos (CPF), a widely used organophosphate pesticide, accumulates in the environment and affects human health. Its neurotoxicity has been extensively studied, and recent research has revealed that it can also lead to abnormal spermatogenesis. However, the factors and molecular mechanisms involved remain unclear. In this study, male Sprague–Dawley rats were gavaged with different concentrations of CPF for 30 days, resulting in a disrupted blood-testis barrier (BTB) and abnormal spermatogenesis. RNA sequencing analysis of Sertoli cells, the primary components of the BTB and key targets of environmental toxins, revealed that ferroptosis-related genes were predominantly among the differentially expressed genes. The expression of ferroptosis-related markers was up-regulated, malondialdehyde and Fe^2+^ levels were elevated, and glutathione levels were reduced in CPF-exposed testicular tissue and its metabolite TCP-exposed Sertoli cells, confirming that CPF exposure triggered ferroptosis in testes and Sertoli cells. Moreover, treatment with ferrostatin-1, a ferroptosis inhibitor, restored Sertoli cell junctional function. Given the important roles of clockophagy and the HIF-1α pathway in ferroptosis, we investigated the activity of clockophagy in testes and Sertoli cells. Unexpectedly, clockophagy activity was found to be enhanced by the significantly reduced expression levels of ARNTL and HIF-1α following CPF and TCP exposure. Notably, *Arntl* knockdown impaired Sertoli cell junctional function. Collectively, these findings strongly indicate that CPF induces ferroptosis in Sertoli cells through activating clockophagy, resulting in the decreased expression of HIF-1α and BTB-associated proteins; this ultimately leads to the disruption of BTB integrity and spermatogenesis dysfunction.

## Introduction

Chlorpyrifos (CPF) is an organophosphorus pesticide that is widely used worldwide.^1^^,^^2^ Owing to its relatively efficient insecticidal ability, it is extensively utilized in agriculture.^3^^,^^4^ However, excessive exposure to CPF has been found to be detrimental to human health. CPF can enter animals and humans through dermal contact, ingestion, and the food chain, leading to its accumulation, metabolism, and subsequent toxic effects; its main metabolite is 3,5,6-trichloro-2-pyridinol (TCP).^5^, ^6^, ^7^ Studies have demonstrated that CPF can cause significant damage to multiple systems or organs, including the nervous system, respiratory system, and placenta, and can even cause obesity.^8^, ^9^, ^10^, ^11^, ^12^ Moreover, recent studies have revealed that CPF can adversely affect male reproduction, as indicated by decreased levels of serum testosterone, follicle-stimulating hormone, and luteinizing hormone, as well as reduced sperm count and quality.^13^ Additionally, CPF has been found to induce structural abnormalities in the seminiferous tubules. An investigation of the mechanisms underlying the CPF-induced impairment of male reproductive function revealed that CPF caused oxidative damage to the testes. The reduced levels of superoxide dismutase, catalase, glutathione peroxidase, and glutathione (GSH) contribute to the observed damage.^14^, ^15^, ^16^ Recent studies have shown that the highly methylated gene PIK3CD might play a role in regulating the epigenetic mechanism of CPF-induced male reproductive toxicology via the Ras, advanced glycation end products (AGE)–receptor of advanced glycation end products (RAGE), and hypoxia-inducible factor-1 (HIF-1) signaling pathways.^17^ Research on the toxic effects of CPF on male reproductive health and its underlying mechanisms can enhance our understanding of this issue. This knowledge can then be utilized to develop effective strategies for preventing and treating CPF toxicity, as well as promoting the responsible use of CPF.

Sertoli cells utilize tight junctions, basal ectoplasmic specialization, gap junctions, and desmosomes to form the blood-testis barrier (BTB), which supports spermatogenesis. The BTB facilitates physical division within the seminiferous epithelium, establishing distinct basal and apical compartments, which not only provides nutrients to germ cells but also prevents toxic substances from entering the tubules and creates an immune privilege site for germ cells.^18^ Recent studies have demonstrated that the antiandrogenic effects of CPF and its primary metabolite, TCP, occur by suppressing the paracrine function of Sertoli cells, which suggests that Sertoli cells are essential for the abnormal spermatogenesis induced by CPF and TCP.^19^ Although scientists have focused on the male reproductive effects of CPF, the toxic effects and specific mechanism underlying the junctional function of Sertoli cells remain unclear and require further exploration.

Ferroptosis is dependent on iron and driven by lipid peroxidation and is officially defined as a unique form of regulated cell death. Ferroptosis is involved in various physiological and pathological processes, including brain injury, muscle injury, liver injury, blood and immune system diseases, vascular injury, retinal injury, spinal motor neuron degeneration, heart injury, lung injury and diseases, kidney injury, and bone development.^20^^,^^21^ Our previous studies revealed that ferroptosis plays a vital role in the impairment of male reproduction.^22^^,^^23^ Autophagy is a degradation process that is evolutionarily conserved across species and plays significant and intricate roles in human health and disease.^24^^,^^25^ Recently, it has been reported that autophagy plays a role in the regulation of ferroptosis.^26^^,^^27^

Clockophagy is a newly discovered type of autophagy that selectively degrades the core circadian clock protein ARNTL (Aryl hydrocarbon receptor nuclear translocator-like, also known as Bmal1), subsequently reducing hypoxia-inducible factor-1 alpha (HIF-1α) expression and inducing ferroptosis.^28^ ARNTL is an important regulator of normal physiological functions in organisms, and its mutation or aberrant expression has been associated with infertility, gluconeogenesis, inflammatory lung disease, lipogenesis, and altered sleep patterns.^29^, ^30^, ^31^, ^32^, ^33^, ^34^, ^35^ Therefore, we hypothesized that the disruption of the BTB and the dysfunction of spermatogenesis induced by CPF may be linked to ferroptosis mediated by clockophagy.

In this study, we use both *in vivo* and *in vitro* approaches to demonstrate that CPF exposure induces spermatogenesis dysfunction by impairing BTB integrity, which is regulated by clockophagy-mediated ferroptosis.

## Materials and methods

### Animals and treatments

Male Sprague–Dawley (SD) rats at postnatal day (PND) 21 were purchased from the Experimental Animal Center of Chongqing Medical University and were housed at the Experimental Animal Center of Children's Hospital of Chongqing Medical University. All animal procedures in this study were approved by the Ethics Committee of the Children's Hospital of Chongqing Medical University (Issue number: CHCMU-IACUC20231012002).

Twenty-one SD rats were randomly divided into three groups and subjected to daily oral gavage with either corn oil (C116023, Aladdin) or CPF (C109843, Aladdin) for 30 consecutive days. There were three groups: a corn oil control group, a CPF group receiving 10 mg/kg body weight (C10), and a CPF group receiving 20 mg/kg body weight (C20). On the day following the last treatment, the rats were euthanized by cervical dislocation. The testes and epididymis were swiftly excised and subjected to a range of measurements.

### Cell culture and treatment

The Sertoli cell line TM4 was cultured in a DMEM/F12 medium enriched with 5 % fetal bovine serum. The cells were incubated in humidified conditions with 5 % CO_2_ at 37 °C. TM4 cells were treated with TCP (33972, Sigma), the major bioactive metabolite of CPF *in vivo*. Ferrostatin-1 (Fer-1; HY-100579, MCE), a ferroptosis inhibitor, was used to suppress ferroptosis in TM4 cells at a concentration of 0.5 μM based on our published article.^22^ Chloroquine (CQ, HY-17589A, MCE), an inhibitor of autophagy, was used to inhibit autophagic flux in TM4 cells, and a concentration of 25 μM was selected according to a published article.^36^ DMSO (D8418, Sigma) was used to dissolve the above reagents. The control group was treated with DMSO. Fer-1 or CQ was combined with TCP for treatment for 24 h.

### CCK-8 assay

TM4 cells were plated in 96-well plates and exposed to various concentrations of TCP for 24 h. Subsequently, 100 μL of 10% CCK-8 reagent (HY–K0301, MCE) was added to each well, and the plates were incubated at 37 °C for 2 h. The optical density was then measured at 450 nm using a spectrophotometer.

### Sperm quality analysis

The epididymis was excised and immersed in 1 mL of 0.9% saline to prepare a suspension. The suspension was subsequently diluted with normal saline at a ratio of 9:1 at 37 °C. The diluted sperm were then counted using the hemocytometer method.^37^^,^^38^

The diluted suspension was spread onto slides, which were then air-dried at room temperature and fixed in methanol for 30 min. The slides were subsequently stained with eosin for 30 min to visualize the sperm morphology.^39^^,^^40^

### Hematoxylin and eosin staining

Paraffin-embedded testis tissue was processed into sections measuring four micrometers in thickness. The tissue sections were deparaffinized and rehydrated using xylene followed by a series of ethanol solutions of decreasing concentrations. Hematoxylin and eosin were used to stain the cell nuclei and cytoplasm, respectively.

### Western blotting

Total protein from the testes and TM4 cells was extracted with RIPA lysis buffer (HY–K1001, MCE) supplemented with 10% protease inhibitor cocktail (HY–K0010, MCE). The experimental procedures are detailed in a previously published article.^41^ In brief, a BCA kit was used to determine the protein concentration, and the loading buffer was used to denature the proteins. The samples were loaded onto SDS-PAGE gels and subsequently transferred onto a PVDF membrane. After being blocked with a rapid blocking buffer, the membranes were incubated with primary antibodies followed by the corresponding secondary antibodies. Images were captured using a chemiluminescence imaging system and analyzed with Image Lab software. The details of the primary antibodies utilized in this study are provided in Table S1.

### Immunofluorescence staining

Four-micrometer paraffin sections (for staining promyelocytic leukaemia zinc finger (PLZF) and stimulated by retinoic acid gene 8 (stra8)) were subjected to antigen retrieval using citrate buffer after deparaffinization and rehydration. TM4 cells were plated in cell coverslips and cultured in the 24-well plates. The cell coverslips and 10-μm frozen sections (for staining BTB-associated proteins and actin-binding proteins) were fixed with 4% paraformaldehyde for 15 min and treated with 0.5% Triton X-100 for 10 min. The procedures were similar to those described in our published article.^41^ In brief, tissue sections or cell coverslips were blocked with 0.5% bovine serum albumin at room temperature for 1 h, followed by overnight incubation with the indicated primary antibodies at 4 °C. After 1 h of incubation with the corresponding fluorescent secondary antibodies at room temperature, the samples were stained with Hoechst 33342 for 30 min and mounted with an anti-fade reagent. Images were acquired with a C2 confocal microscope (Nikon). The details of the primary antibodies utilized in this study are provided in Table S1.

### BTB integrity assay

EZ-Link SulfoNHS-LC-Biotin (21335, Thermo) was used to determine the BTB integrity according to a published method.^42^ After the rats were anesthetized, a 1-cm incision was made in the scrotum to expose the testes, and 100 μL of 10 mg/mL EZ-Link Sulfo–NHS–LC-Biotin was gently injected under the tunica albuginea using a 28-gauge needle. After 30 min, the testes were immediately removed and snap-frozen in liquid nitrogen. The 10-μm sections were fixed with 4% paraformaldehyde for 15 min and incubated with an Alexa Fluor 555-streptavidin conjugate for 30 min. The nuclei were stained with Hoechst 33342 for 30 min after washing with phosphate buffer saline. The images were acquired with a C2 confocal microscope (Nikon) after the sections were mounted with an anti-fade reagent.

### ROS, MDA, GSH, and ferrous ion assays

The levels of reactive oxygen species (ROS; E-BC-K138-F, Elabscience), malondialdehyde (MDA; S0131S, Beyotime), GSH (E-BC-K030-M, Elabscience), and ferrous iron (E-BC-K773-M, Elabscience) were measured using specific assay kits. The experimental procedures were conducted following the instructions provided with each respective kit.

### Calcein-AM/propidium iodine staining

The number of dead cells was determined with a calcein-AM/propidium iodine assay kit (C2015M, Beyotime). The experimental steps were performed according to the corresponding kit instructions. The images were acquired by a fluorescence microscope (Nikon).

### Ferrous iron and lipid peroxidation detection

The levels of divalent iron ions in TM4 cells were assessed with the FerroOrange (36104, CST). C11 BODIPY 581/591 (D3861, Thermo) was used to assess lipid peroxidation in TM4 cells. The detection procedures were carried out following the instructions provided with each respective kit. The cells were incubated with Hoechst 33342 for 30 min. Ferrous iron and lipid peroxidation images were acquired with a C2 confocal microscope (Nikon).

### RNA sequencing

The RNA sequencing of three DMSO-treated and three TCP-treated TM4 cell samples was performed by Gene Denovo Biotech Ltd., Guangzhou, China. Total RNA was extracted using a TrIzol reagent kit (Invitrogen, Carlsbad, CA, USA) following the manufacturer's instructions. After RNA extraction and quality evaluation, the mRNA was enriched via oligo (dT) beads. The enriched mRNA was then fragmented into short pieces with fragmentation buffer and reverse-transcribed into cDNA using the NEBNext Ultra RNA Library Prep Kit for Illumina (NEB #7530, New England Biolabs, Ipswich, MA, USA). The purified double-stranded cDNA fragments were end-repaired, an A base was added, and the fragments were ligated to Illumina sequencing adapters. The ligation products were purified using AMPure XP beads (1.0×) and then subjected to PCR amplification. The final cDNA library was sequenced on the Illumina NovaSeq 6000 platform.

### Autophagic flux assay

The autophagic flux of TM4 cells was determined with the Ad-mCherry-GFP-LC3B (C3011, Beyotime). TM4 cells were plated in 24-well plates and treated with DMSO, TCP, or Fer-1. Then, the TM4 cells were infected with Ad-mCherry-GFP-LC3B for 24 h (multiplicity of infection = 30). The cells were incubated with Hoechst 33342 for 30 min. Autophagic flux images were acquired with a C2 confocal microscope (Nikon).

### LysoTracker and LysoSensor staining

LysoSensor Green DND-189 (L7535, Thermo) and LysoTracker Green DND-26 (L7526, Thermo) were used to determine the pH and the number of lysosomes, respectively. Hoechst 33342 was used to stain the cell nucleus after LysoSensor and LysoTracker staining. The images were acquired with a C2 confocal microscope (Nikon).

### siRNA-mediated *Arntl* knockdown

*Arntl* siRNA was purchased from Beijing Tsingke Biotech (China). The target sequence of the *Arntl* siRNA used *in vitro* was GAUAACGACCAAGGAUCAA. Lipo6000 Transfection Reagent (C0526, Beyotime) was used to transfect TM4 cells. After transfection for 4 h, the medium was replaced with a fresh culture medium, and the cells were incubated for an additional 72 h. The cells were subsequently collected for further experiments.

### Statistical analyses

To compare two groups, we utilized either an unpaired *t*-test or Welch's *t*-test, depending on the results of the F-test for homogeneity of variance. When analyzing three or more groups, we conducted a one-way analysis of variance (ANOVA), followed by Tukey's test for pairwise comparisons and Dunnett's test to assess differences relative to a control group. The statistical analyses were performed using GraphPad Prism 9 software, with the significance level set at *p* < 0.05.

## Results

### The toxic effects of CPF on spermatogenesis function

**Figure 1:**
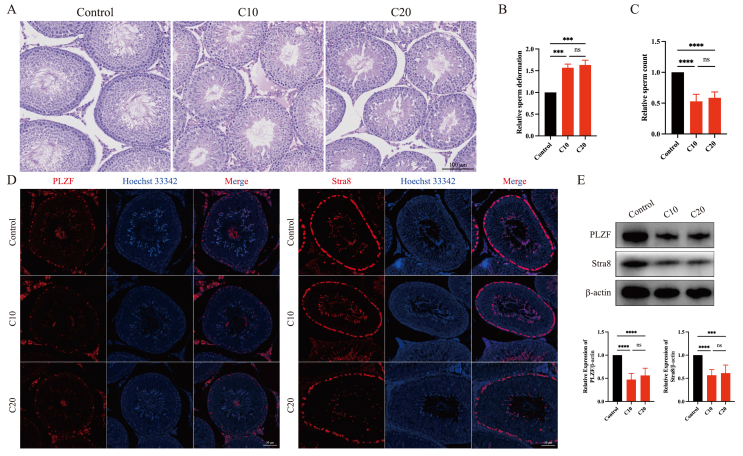
The toxic effects of chlorpyrifos exposure on spermatogenesis dysfunction. **(A)** The histological changes in the testes of rats in the three different groups. **(B)** The sperm deformation rate. **(C)** The number of sperm. **(D)** Immunofluorescence analysis of the expression of PLZF and Stra8 in the rat testes. **(E)** The protein expression levels of PLZF and Stra8 in the rat testes by Western blot. ∗∗∗*p* < 0.001 and ∗∗∗∗*p* < 0.0001.

First, the toxic effects of CPF on testes were verified via hematoxylin-eosin staining. The seminiferous tubules exhibited vacuoles and fissures in the testes exposed to CPF (Fig. 1A). Moreover, CPF exposure led to a much greater degree of sperm deformation and a lower number of sperm (Fig. 1B, C). Additionally, we examined the expression of PLZF and Stra8 to assess the self-renewal of spermatogonia and the meiosis of spermatocytes. Immunofluorescence staining (Fig. 1D) and western blotting (Fig. 1E) revealed that CPF exposure resulted in decreased expression of these two markers in the testis. These results suggested that CPF impaired spermatogenesis in SD rats.

### CPF exposure disrupted BTB integrity

**Figure 2:**
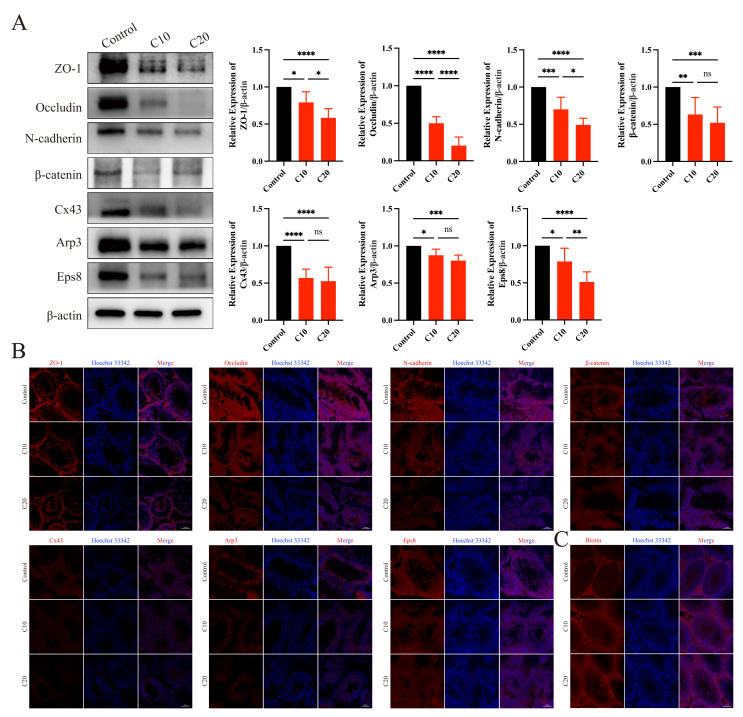
Chlorpyrifos exposure disrupted the blood-testis barrier integrity. **(A)** The expression of blood-testis barrier-associated proteins and actin-binding proteins in the three different groups. **(B)** The localization of blood-testis barrier-associated proteins and actin-binding proteins in the three different groups. **(C)** Blood-testis barrier integrity analysis. ∗*p* < 0.05, ∗∗*p* < 0.01, ∗∗∗*p* < 0.001, and ∗∗∗∗*p* < 0.0001; ns, not significant.

Considering that the integrity of the BTB is essential for normal spermatogenesis,^18^ we speculated that CPF exposure may impair the BTB integrity. After CPF exposure, the expression levels of tight junction proteins (ZO-1 and Occludin), basal ectoplasmic specialization proteins (N-cadherin and β-catenin), and gap junction protein (Cx43) were obviously reduced (Fig. 2A). However, the locations of these proteins did not change; they were still around the basal compartment (Fig. 2B). Moreover, a biotin tracing assay showed that the BTB integrity was disrupted after CPF treatment (Fig. 2C).

Actin-binding proteins (ABPs) regulate bundling and branching changes in actin filaments to “open” and “close” the BTB.^43^ Actin-related protein 2/3 (Arp2/3) promotes branching changes in bundled actin, whereas epidermal growth factor receptor pathway substrate 8 (Eps8) has the opposite effect, facilitating bundling changes in branched actin.^44^ After CPF exposure, the expression levels of Arp3 and Eps8 decreased significantly (Fig. 2A) without notable translocation (Fig. 2B). These results indicated that CPF caused abnormal spermatogenesis by compromising the integrity of the BTB.

### TCP exposure compromised the Sertoli cell junctional function

**Figure 3:**
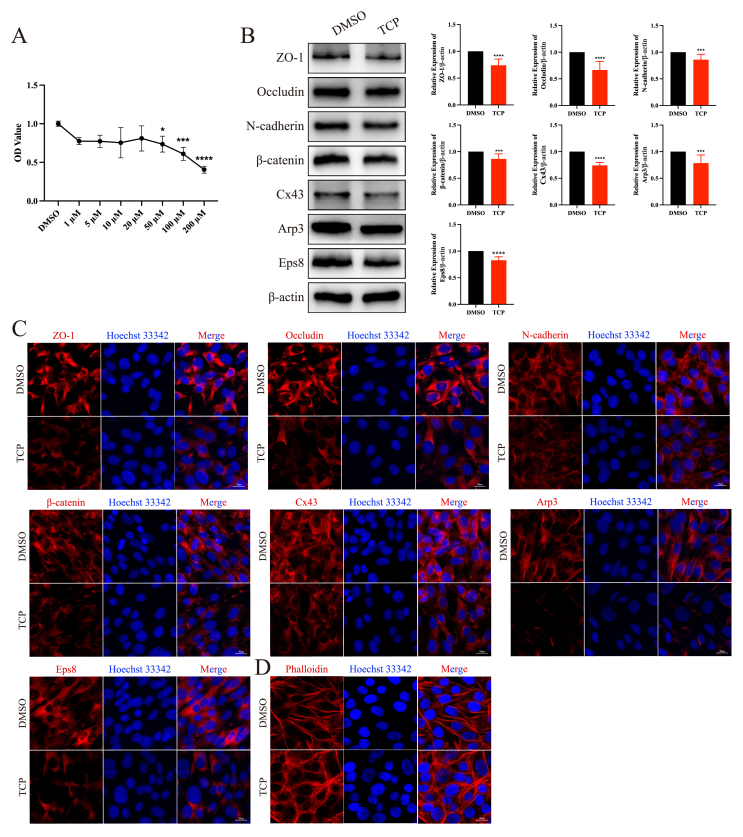
TCP exposure disrupted the junctional function of Sertoli cells. **(A)** CCK-8 assays. **(B)** The expression of blood-testis barrier-associated proteins and actin-binding proteins in Sertoli cells following TCP exposure. **(C)** The localization of blood-testis barrier-associated proteins and actin-binding proteins in Sertoli cells following TCP exposure. **(D)** The cytoskeleton organization in Sertoli cells following TCP exposure. ∗*p* < 0.05, ∗∗*p* < 0.01, ∗∗∗*p* < 0.001, and ∗∗∗∗*p* < 0.0001.

Since the BTB is formed by tight junctions, basal ectoplasmic specialization, gap junctions, and desmosomes between adjacent Sertoli cells, we exposed Sertoli cells to TCP for 24 h. CCK-8 assays revealed that the viability of Sertoli cells obviously decreased with increasing TCP concentration; we selected 50 μM TCP for subsequent experiments based on these results (Fig. 3A). Consistent with the *in vivo* results, the expression levels of ZO-1, Occludin, N-cadherin, β-catenin, Cx43, Arp3, and Eps8 were reduced obviously (Fig. 3B), and the localization of these proteins did not obviously change (Fig. 3C). Notably, TCP exposure led to disorganization of the cellular actin cytoskeleton (Fig. 3D). The results showed that TCP exposure disrupted Sertoli cell junctional function.

### TCP exposure disrupted junctional function by triggering ferroptosis in Sertoli cells

**Figure 4:**
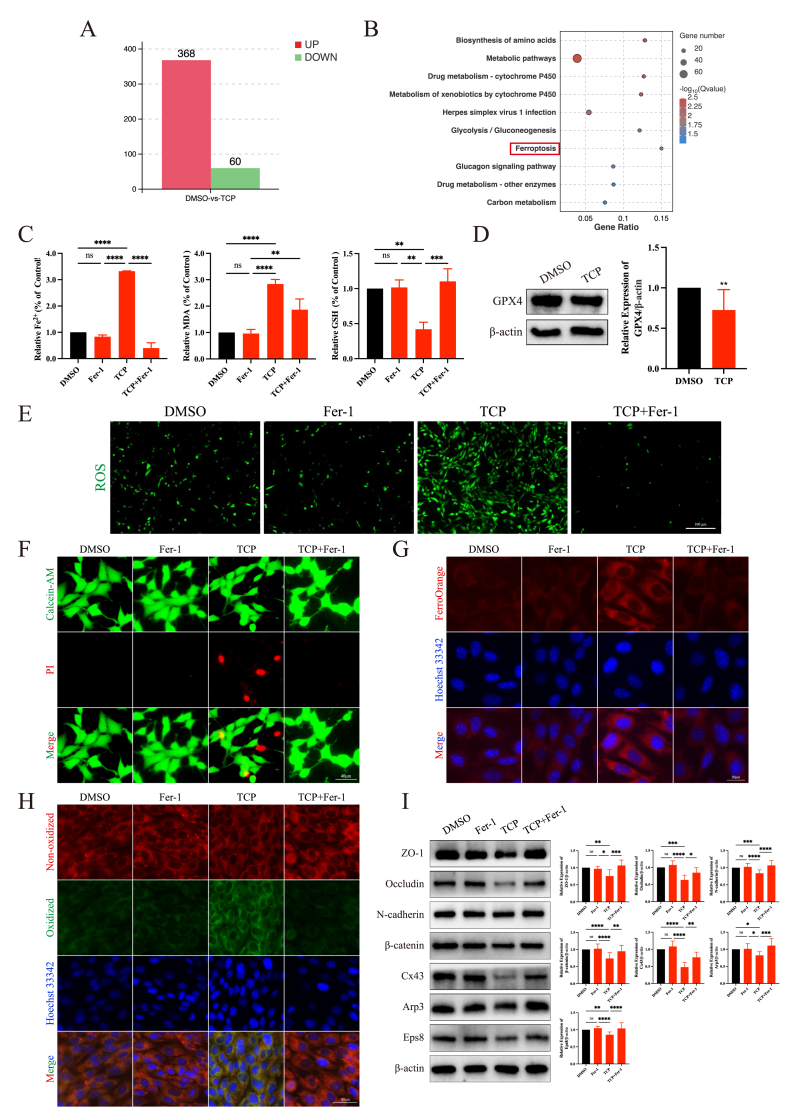
TCP exposure disrupted junctional function by triggering ferroptosis in Sertoli cells. **(A)** The differentially expressed genes between the DMSO and TCP groups. **(B)** The results of the KEGG analysis. **(C)** The levels of Fe^2+^, malondialdehyde (MDA), and glutathione (GSH) in Sertoli cells in the four different groups. **(D)** The protein expression level of GPX4 after TCP exposure. **(E)** Reactive oxygen species (ROS) generation in the four different groups. **(F)** Calcein-AM/propidium iodine (PI) staining showed that ferrostatin-1 (Fer-1) treatment reversed the cell death induced by TCP exposure. **(G)** FerroOrange staining revealed that Fer-1 treatment decreased the level of ferrous ions induced by TCP exposure. **(H)** Fer-1 treatment reduced the level of lipid peroxidation induced by TCP exposure. **(I)** Fer-1 treatment reversed the decrease in the protein expression levels of blood-testis barrier-associated proteins after TCP exposure. ∗*p* < 0.05, ∗∗*p* < 0.01, ∗∗∗*p* < 0.001, and ∗∗∗∗*p* < 0.0001; ns, not significant.

To better explore the underlying mechanisms, RNA sequencing was performed on Sertoli cells exposed to TCP. A total of 368 genes were up-regulated, whereas 60 genes were down-regulated (Fig. 4A). Furthermore, Kyoto Encyclopedia of Genes and Genomes (KEGG) analysis revealed that the differentially expressed genes were enriched primarily in metabolic pathways, glycolysis, and ferroptosis (Fig. 4B). Thus, we speculated that TCP may impair the Sertoli cell junctional function by inducing ferroptosis.

Consistent with our speculation, in the TCP-treated Sertoli cells, the levels of Fe^2+^ and MDA were increased (Fig. 4C); the levels of GSH (Fig. 4C) and glutathione peroxidase 4 (GPX4) were reduced (Fig. 4D); and the ROS level (Fig. 4E), the number of dead cells (Fig. 4F), and the lipid peroxidation level (Fig. 4H) were enhanced. Additionally, FerroOrange staining showed that TCP exposure increased ferrous iron levels (Fig. 4G). Fer-1 treatment subsequently reversed TCP-induced Sertoli cell ferroptosis; normalized the levels of ferrous iron, MDA, GSH, ROS, and lipid peroxidation; and decreased the number of dead cells (Fig. 4C–H). These results demonstrated that TCP induced Sertoli cell ferroptosis.

To elucidate the relationship between Sertoli cell ferroptosis and junctional function, we examined the expression levels of BTB-associated proteins in Sertoli cells after treatment with Fer-1. Western blotting showed that Fer-1 restored the expression levels of junctional proteins to normal levels (Fig. 4I), suggesting that TCP disrupted the Sertoli cell junctional function by inducing ferroptosis.

### TCP exposure induced Sertoli cell ferroptosis by activating clockophagy

**Figure 5:**
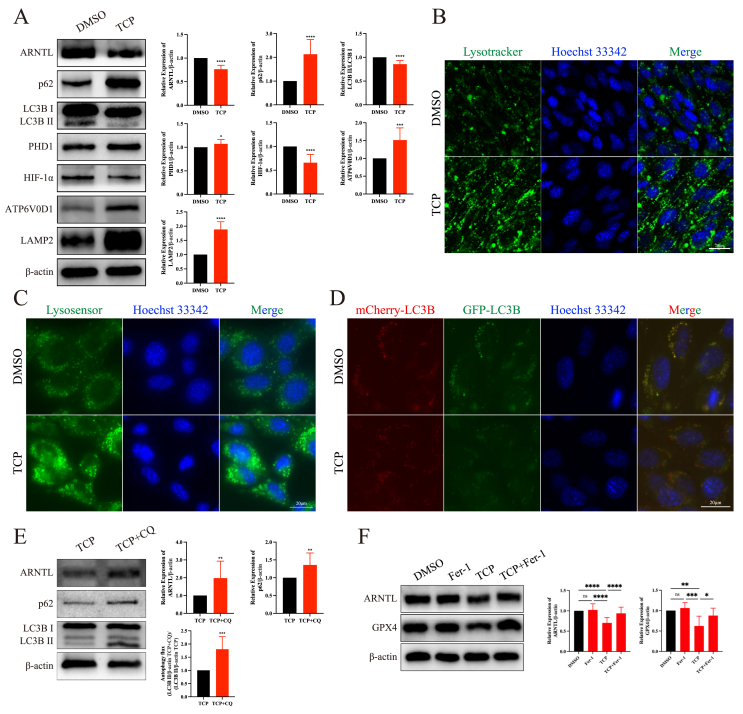
TCP exposure enhanced clockophagy activity and lysosomal function in Sertoli cells. **(A)** The protein expression levels of markers of clockophagy and lysosomal function after TCP exposure. **(B)** LysoTracker staining showed that TCP exposure increased the number of lysosomes. **(C)** LysoSensor staining showed that TCP exposure enhanced lysosomal function. **(D)** TCP exposure enhanced autophagic flux in Sertoli cells. **(E)** Chloroquine (CQ) treatment inhibited clockophagy-mediated ARNTL degradation. **(F)** Ferrostatin-1 (Fer-1) treatment rescued the protein expression levels of ARNTL and GPX4, which decreased after TCP exposure. ∗*p* < 0.05, ∗∗*p* < 0.01, ∗∗∗*p* < 0.001, and ∗∗∗∗*p* < 0.0001; ns, not significant.

Because HIF-1α plays an important role in ferroptosis,^45^^,^^46^ we next examined the expression of HIF-1α and found that its expression level was significantly reduced in Sertoli cells after exposure to TCP (Fig. 5A). Clockophagy, a newly identified type of selective autophagy, plays a role in triggering ferroptosis by degrading ARNTL and causing subsequent instability of HIF-1α. Given the reduction in HIF-1α expression, we speculated that clockophagy may be involved in regulating TCP-induced ferroptosis in Sertoli cells. Unsurprisingly, the protein expression levels of ARNTL and light chain 3 beta (LC3B) II/I decreased, and the expression levels of p62 and prolyl hydroxylase 1 (PHD1) increased in Sertoli cells after TCP exposure (Fig. 5A). Because the ratio of LC3B-II (a conjugated form) to LC3B–I (an unconjugated form) was decreased and autophagy is a protein degradation process that relies on lysosomes, we speculated that TCP could enhance lysosomal function to degrade autophagosomes. The levels of ATPase H^+^ transporting V0 subunit D1 (ATP6V0D1) and lysosome-associated membrane protein-2 (LAMP2), which are markers of lysosomal function, were increased after TCP exposure (Fig. 5A). Furthermore, LysoTracker and LysoSensor staining revealed that TCP increased the number of lysosomes and enhanced lysosomal function (Fig. 5B, C). Additionally, we used Ad-mCherry-GFP-LC3B to assess autophagic flux. After TCP exposure, the intensity of green fluorescence decreased dramatically (Fig. 5D). Compared with treatment with TCP alone, treatment with CQ inhibited LC3B II/I degradation (Fig. 5E). The results showed that TCP exposure activated clockophagy.

We next investigated whether the pharmacological blockade of ferroptosis inhibited the down-regulation of ARNTL expression induced by TCP. Fer-1 reversed the TCP-induced reduction of ARNTL and GPX4 expression in Sertoli cells (Fig. 5F), indicating that TCP exposure induced Sertoli cell ferroptosis by activating clockophagy.

### ARNTL knockdown impaired the junctional function of Sertoli cells

**Figure 6:**
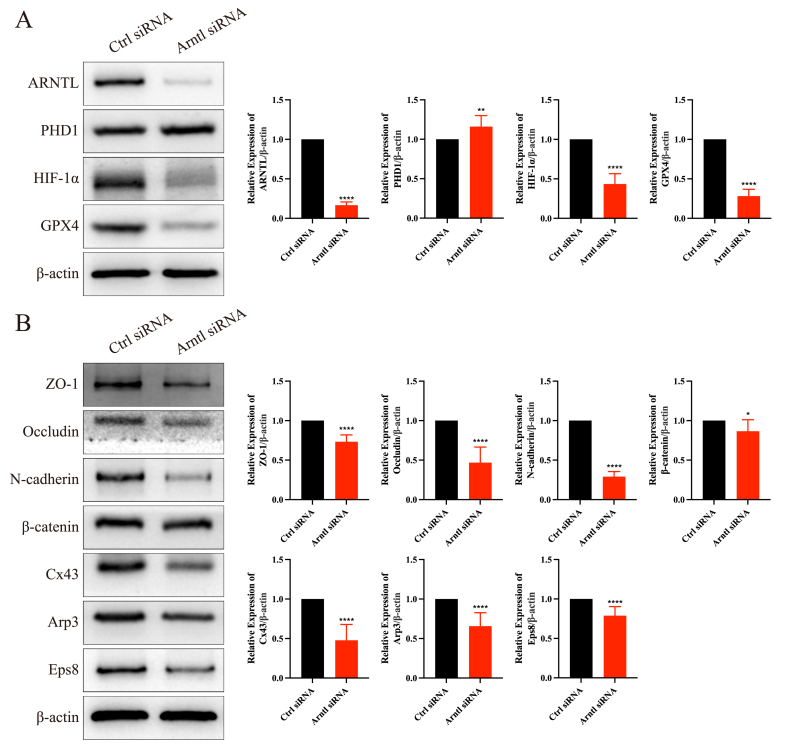
ARNTL regulated the junctional function of Sertoli cells. **(A)** The protein expression levels of ARNTL and its downstream genes after *Arntl* knockdown in Sertoli cells. **(B)** The protein expression levels of blood-testis barrier-associated proteins after *Arntl* knockdown in Sertoli cells. ∗*p* < 0.05, ∗∗*p* < 0.01, and ∗∗∗∗*p* < 0.0001.

Considering that TCP exposure significantly reduced ARNTL expression, we knocked down ARNTL expression to further elucidate the role of ARNTL-mediated clockophagy in regulating Sertoli cell junctional function. The knockdown efficiency of ARNTL was approximately 80% (Fig. 6A). ARNTL knockdown increased the expression of PHD1, decreased the expression of HIF-1α and GPX4 (Fig. 6A), and reduced the expression of BTB-associated proteins (Fig. 6B). These results suggest that ARNTL is crucial for clockophagy-mediated ferroptosis and maintaining Sertoli cell junctional function.

### CPF-induced ferroptosis via clockophagy in the testes

**Figure 7:**
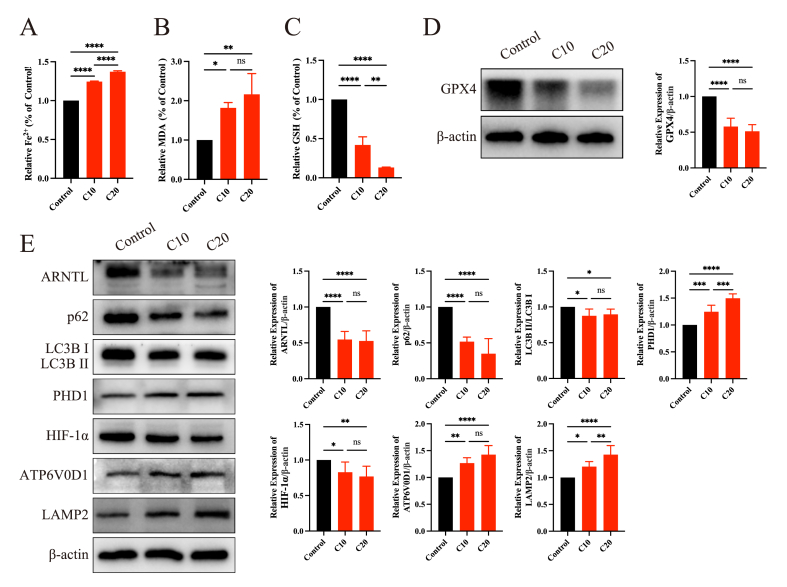
Chlorpyrifos exposure promoted ferroptosis in the testes by activating clockophagy. **(A)** The level of Fe^2+^ in the three different groups. **(B)** The level of malondialdehyde (MDA) in the three different groups. **(C)** The level of glutathione (GSH) in the three different groups. **(D)** The protein expression level of GPX4 in the three different groups. **(E)** The protein expression levels of markers of clockophagy and lysosomal function in the three different groups. ∗*p* < 0.05, ∗∗*p* < 0.01, ∗∗∗*p* < 0.001, and ∗∗∗∗*p* < 0.0001; ns, not significant.

We examined the ferroptosis level and clockophagy activity in the testes after CPF exposure. The ferroptosis level in the testes was consistent with the *in vitro* results. After exposure to CPF, the levels of Fe^2+^ (Fig. 7A) and MDA (Fig. 7B) increased, and the level of GSH (Fig. 7C) and the expression level of GPX4 (Fig. 7D) markedly decreased. These results indicated that CPF exposure promoted ferroptosis in the testes. Moreover, clockophagy activity in CPF-treated testes was significantly increased; the expression levels of ARNTL, p62, LC3B II/I, and HIF-1α were dramatically reduced; and the expression levels of PHD1, ATP6V0D1, and LAMP2 were enhanced (Fig. 7E), suggesting that CPF exposure activated clockophagy in testes.

## Discussion

CPF is a widely used organophosphorus pesticide on a global scale. However, the excessive use of CPF has been proven to lead to environmental burdens in certain regions and populations. Studies have shown that CPF impairs male reproduction through a decrease in sperm count and quality and the abnormal secretion of sex hormones. TCP is the major metabolite of CPF and has been reported to be potentially more toxic than CPF.^7^ The BTB is the primary target of environmental toxicants; however, no studies have focused on the junction function of Sertoli cells. Understanding whether and how CPF affects the junctional function of Sertoli cells is of significant importance.

It has been demonstrated that exposure to toxicants during the prepubertal stage has detrimental effects on the development and maturation of the male reproductive system.^47^, ^48^, ^49^ These effects can persist into adulthood and lead to abnormal spermatogenesis. Additionally, the BTB completes assembly by PND 18–21 in rats; therefore, we administered CPF to male SD rats from PND 21 to explore the relationship between BTB integrity and abnormal spermatogenesis induced by CPF exposure.^50^ Unexpectedly, prepubertal exposure to CPF resulted in abnormal testicular morphology, decreased sperm count and quality, and BTB integrity impairment, suggesting that CPF resulted in abnormal spermatogenesis by disrupting BTB integrity.

According to the RNA sequencing results, the differentially expressed genes were enriched mainly in the ferroptosis pathway. We subsequently focused on ferroptosis to investigate whether it played a vital role in regulating the junctional function of Sertoli cells. In this study, we confirmed that CPF and TCP promoted ferroptosis *in vivo* and *in vitro*, respectively. Furthermore, treatment with Fer-1 suppressed the TCP-induced ferroptosis and impairment of junctional function in Sertoli cells, revealing that ferroptosis was the main factor involved in the disruption of the BTB induced by CPF. Recent studies have discovered that ferroptosis can induce inflammation in Leydig cells and the release of pro-inflammatory cytokines in the spinal cords of rats with inflammatory pain.^51^^,^^52^ Sertoli cells are capable of secreting several cytokines, such as IL-1α, TNF-α, and TGF-β3, to regulate the opening of the BTB.^53^ Therefore, whether CPF-induced ferroptosis in Sertoli cells promotes the secretion of these cytokines, thereby impairing the BTB integrity, still needs to be explored.

Recently, ferroptosis has been identified as an autophagy-dependent form of cell death.^54^ Clockophagy, involving the selective degradation of the core circadian clock protein ARNTL, suppresses the expression of HIF-1α and promotes ferroptosis. A previous study demonstrated that CPF exposure reduced HIF-1α expression,^55^ and we hypothesized that clockophagy may be involved in CPF-induced ferroptosis. Consistent with our hypothesis, clockophagy activity was enhanced after CPF exposure. ARNTL has various critical roles in maintaining human health, and *Arntl* knockout induces infertility in male mice.^56^, ^57^, ^58^ Circadian clock proteins regulate mitochondrial dynamics, including oxidative phosphorylation and ATP production, which are crucial for maintaining normal physiology,^59^ and mitochondria play important roles in the regulation of ferroptosis. The BTB is not quiescent; it “opens” and “closes” at the special stages through the dynamic states of “bundled” and “unbundled” actin microfilaments. Energy is required for maintaining the BTB dynamics.^18^ Thus, ARNTL may be involved in BTB dynamics through mitochondria dysfunction-mediated ferroptosis.

LC3B is a marker gene for autophagosomes; it is a key protein involved in the formation of autophagosomes during autophagy and is commonly used to label and detect autophagic activity.^60^^,^^61^ However, the LC3B II/I ratio decreased in CPF-exposed testes and TCP-exposed Sertoli cells. During the process of autophagy, autophagosomes fuse with lysosomes, forming structures known as autolysosomes. Within autolysosomes, the contents of autophagosomes are enzymatically hydrolyzed and degraded, allowing for the clearance and recycling of intracellular components that are deemed unnecessary or damaged.^25^ Thus, we believe that the reduction in LC3B II/I was due to enhanced lysosomal degradation. LysoTracker and LysoSensor staining demonstrated that TCP exposure increased the number and function of lysosomes, suggesting that CPF and TCP increased autophagic flux *in vivo* and *in vitro*, respectively. Moreover, Ad-mCherry-GFP-LC3B infection further confirmed the increased autophagic flux in Sertoli cells.

Notably, the changes in p62 expression levels were different between TCP-treated Sertoli cells and CPF-treated testes. This may be due to the presence of other types of cells in the testicular tissues. Moreover, p62 not only plays a role in regulating autophagy but also promotes the transcription of antioxidant enzymes by regulating NRF2 expression.^62^ Therefore, this could be the reason for the inconsistency in p62 expression *in vivo* and *in vitro*. Additionally, the expression level of p62 alone is insufficient to assess autophagic activity.^63^

To further clarify the relationship between ARNTL and the junctional function of Sertoli cells, we knocked down *Arntl* expression in Sertoli cells and found that Hif-1α expression was markedly decreased. Following *Arntl* knockdown, the expression levels of BTB-associated proteins and ABPs were markedly decreased, suggesting that the junctional function of Sertoli cells was disrupted. The results not only confirmed the crucial role of ARNTL in maintaining Sertoli cell junctional function but also demonstrated that CPF impaired BTB integrity and led to spermatogenesis dysfunction by activating clockophagy.

Our study provides a new perspective that CPF impairs the junctional function of Sertoli cells through clockophagy-mediated ferroptosis. However, this study has several limitations. First, the CPF exposure levels are higher than realistic human doses; we plan to study the toxic effects of low-dose, long-term CPF exposure, which is consistent with realistic exposure levels. Second, we only used prepubertal male SD rats to study the reproductive toxicity of CPF. It is important to include male SD rats from various age groups to gain a more comprehensive understanding of the toxic effects of CPF on male reproduction. Third, our research focused on the toxic effects of CPF on the junctional function of Sertoli cells. In addition to the junctional function, the roles of secretion and phagocytosis are also crucial for spermatogenesis and should be explored in future studies. Therefore, much work remains to be done to better understand the impacts of CPF on Sertoli cells and to guide its use effectively.

In conclusion, our findings demonstrate that CPF exposure compromises the BTB integrity via clockophagy-mediated ferroptosis, which leads to abnormal spermatogenesis. Our findings provide novel insights into understanding the mechanisms of CPF-induced male infertility.

## CRediT authorship contribution statement

**Yan Fu:** Writing – original draft, Visualization, Investigation. **Xu Huang:** Software, Methodology, Investigation. **Siyuan Wang:** Software, Methodology, Investigation. **Qitong Guo:** Software, Methodology, Investigation. **Yuhao Wu:** Visualization, Data curation. **Xiangqin Zheng:** Methodology, Data curation. **Junke Wang:** Visualization, Data curation. **Shengde Wu:** Validation, Methodology. **Lianju Shen:** Writing – review & editing, Project administration, Funding acquisition, Conceptualization. **Guanghui Wei:** Writing – review & editing, Supervision, Funding acquisition.

## Funding

This study was supported by the 10.13039/501100001809National Natural Science Foundation of China (No. 81801521), the 10.13039/501100002865Chongqing Science and Technology Bureau of China (No. CSTB2022NSCQ-LZX0019, 2022ZDXM033), and the 10.13039/501100007957Chongqing Municipal Education Commission of China (No. KJQN202200410).

## Conflict of interests

The authors declare that they have no known competing financial interests or personal relationships that could have appeared to influence the work reported in this paper.
