# Supplementary material for: Chlorpyrifos induces spermatogenic dysfunction via ferroptosis in Sertoli cells
Source: Genes Dis. 2025 Mar 14;12(5):101601. doi: 10.1016/j.gendis.2025.101601 (PMC12242442; doi:10.1016/j.gendis.2025.101601)
Supplement: Multimedia component 1 [file mmc1.docx]

**Supplementary Table 1. Antibodies utilized for different experiments in this study**

| Antibody | Host Species | Vendor | Catalog Number | WB | IF |
| --- | --- | --- | --- | --- | --- |
| β-actin | Mouse | ZSGB | TA-09 | 1:1000 |  |
| PLZF | Mouse | Santa Cruz | sc-28319 |  | 1:10 |
| Stra8 | Rabbit | Abcam | ab49602 |  | 1:200 |
| PLZF | Rabbit | Abclonal | A21185 | 1:2000 |  |
| Stra8 | Mouse | Proteintech | 68071-1-Ig | 1:1000 |  |
| ZO-1 | Rabbit | Invitrogen | 61-7300 | 1:1000 | 1:100 |
| Occludin | Rabbit | Abcam | ab216327 | 1:1000 | 1:100 |
| N-cadherin | Rabbit | Proteintech | 22018-1-AP | 1:1000 | 1:100 |
| β-catenin | Rabbit | Proteintech | 51067-2-AP | 1:1000 | 1:100 |
| Cx43 | Rabbit | Abclonal | A11752 | 1:1000 | 1:100 |
| Arp3 | Rabbit | Abclonal | A4514 | 1:1000 | 1:100 |
| Eps8 | Rabbit | CST | 43114 | 1:1000 | 1:100 |
| LAMP2 | Rabbit | Abclonal | A0593 | 1:1000 |  |
| Phalloidin |  | Abclonal | RM02835 |  | 1:100 |
| GPX4 | Rabbit | Zenbio | 381958 | 1:1000 |  |
| ARNTL | Rabbit | proteintech | 14268-1-AP | 1:1000 |  |
| p62 | Rabbit | CST | 23214 | 1:1000 |  |
| PHD1 | Rabbit | Zenbio | 380721 | 1:1000 |  |
| HIF-1α | Rabbit | CST | 36169 | 1:1000 |  |
| LC3B | Rabbit | Abclonal | A19665 | 1:1000 |  |
| Peroxidase-AffiniPure Goat Anti-Rabbit IgG (H+L) |  | Jackson | 111-035-003 | 1:20000 |  |
| Peroxidase-AffiniPure Goat Anti-Mouse IgG (H+L) |  | Jackson | 115-035-003 | 1:20000 |  |
| Cy3-conjugated Affinipure Goat Anti-Mouse IgG |  | Proteintech | SA00009-1 |  | 1:200 |
| Cy3-conjugated Affinipure Goat Anti-Rabbit IgG |  | Proteintech | SA00009-2 |  | 1:200 |

Abbreviations: WB, western blot; IF, immunofluorescence.
